# Parents' experiences of handling oral anticancer drugs at home: ‘It all falls on me …’

**DOI:** 10.1111/jep.13737

**Published:** 2022-08-04

**Authors:** Ranaa Akkawi El Edelbi, Staffan Eksborg, Ulrika Kreicbergs, Malin Lövgren, Klara Wallén, Jennie Ekman, Synnöve Lindemalm

**Affiliations:** ^1^ Department of Women's and Children's Health, Childhood Cancer Research Unit Karolinska Institutet Stockholm Sweden; ^2^ Division of Pediatrics, Karolinska University Hospital Astrid Lindgren Children's Hospital Stockholm Sweden; ^3^ Department of Health Care Sciences, Palliative Research Centre Ersta Sköndal Bräcke University College Stockholm Sweden; ^4^ Advanced Pediatric Home Care Karolinska University Hospital Stockholm Sweden; ^5^ Department of Clinical Sciences, Intervention and Technology (CLINTEC) Karolinska Institutet Stockholm Sweden

**Keywords:** experiences, handling, home, oral anticancer drugs, parents

## Abstract

**Aim:**

The aim of this study was to describe the experiences of parents handling oral anticancer drugs in a home setting.

**Methods:**

Parents of children with cancer were recruited from a paediatric oncology ward in Sweden to participate in an interview. The interviews were transcribed verbatim and subjected to qualitative content analysis.

**Results:**

We found the following categories and subcategories: parents’ views on the provided information—*lack of, too little or contradictory information, and parents’ preferences for information delivery*; safety over time; correct drug dose; and drug administration. As time passed, most parents adapted to their child's illness, felt safer and found it easier to take in and process any given information. Parents preferred information in different formats (written, movie clips and orally) and in their mother tongue. Many parents were aware of the importance of giving an accurate dose to their child and described the process of drug administration as overwhelming.

**Conclusion:**

Parents need to be provided with accurate, timely, nonconflicting and repeated information—in different forms and in their mother tongue—on how to handle oral anticancer drugs at home.

**Abbreviations**
HCP: Health Care Professional
OAD: Oral Anticancer Drug
PPE: Personal Protective Equipment

## 1 INTRODUCTION

Parenting a child with cancer is emotionally overwhelming.^1^, ^2^, ^3^ Psychological problems such as stress and depression are more common in parents of children with cancer than in those of healthy children.^4^ To handle the medication and care of children in a safe and optimal way at home, parents must obtain knowledge and skills while adjusting to the fact that their child has cancer.^5^, ^6^ To attain and understand the information provided can be likened to learning a new language.^7^, ^8^, ^9^

Parents use different sources to acquire answers when they lack information, for example, some browse the internet and some use other parents as a source of information. Seeking answers give parents a sense of control and decreases their anxiety.^7^, ^10^, ^11^, ^12^

At the beginning of the cancer treatment period, parents can have difficulties hearing and understanding the complex information given to them.^1^, ^13^, ^14^, ^15^ As treatment proceeds, their comprehension of having a child with cancer seems to become easier.^16^

Parents feel frustrated when they receive inconsistent information and become tired of seeking information on their own to help them care for their child at home.^17^ To feel safe and secure, parents need to have the right information at the right time with good continuity.^2^, ^18^, ^19^ They can feel strengthened when information is repeated, especially those who do not even remember that they have had a dialogue with the health care professionals (HCPs).^15^, ^16^ The information needs to be adjusted for the parents individually and presented in different formats: written, movie clips and orally.^5^

Before 2009, the local pharmacies in Sweden used to compound suitable child‐friendly formulae from solid forms such as tablets or capsules. The compounding of oral anticancer formulae was discontinued in 2009 to minimize the risk of exposure for the pharmacy employees. The child's parents are now responsible for handling oral anticancer drugs (OADs), that is, facilitating drug administration and/or giving the correct dose, at home. Most often without child‐friendly formulations.

The aim of this study was to describe the experience of parents handling OADs in the home setting.

## 2 METHOD

### 2.1 Study design

This interview study included parents of children with cancer and was conducted between March and May 2019.

### 2.2 Participants

Parents were selected through purposeful sampling that also took into consideration the OAD form, the manipulation method and the duration of treatment. We excluded parents who were not able to communicate in Swedish and were not provided with an interpreter. The recruitment took place at one Swedish paediatric oncology center between March and May 2019. In total 19 parents were recruited and accepted to participate in the study. However, one non‐Swedish‐speaking parent was excluded. Thus, totally 18 parents were included in the study. This number was considered sufficiently large to ensure enough information‐rich cases to describe parents’ views on the information provided.^20^ All participants were parents of paediatric patients with acute lymphoblastic leukaemia or brain tumours who were treated with OADs at home; see Table 1.

**Table 1:** Demographic data of the children and participating parents

Age of child (years)	5.5 (2–14)
Number of patients	18
Female sex	55.5% (10)
Number of parents	19
Female sex	78.9% (15)
Patients receiving OADs for <1 year	72.2% (13)
Patients receiving OADs for >1 year	27.7% (5)
Diagnosis	
Leukaemia	55.5% (10)
Brain tumour	44.4% (8)
Parents with non‐Swedish background	26.3% (5)

*Note*: Data are expressed as median (range) or percentages (%).

Abbreviation: OAD, oral anticancer drug.

### 2.3 Interviews

The interviews took place in a room in a paediatric oncology ward. While the parents were interviewed, the children were playing in the ward, staying with the other parent or present during the interview. The first author (R. A.) conducted all the interviews with each of the parents using a semi‐structured interview guide (Supporting Information: Appendix 1). The guide included questions such as: ‘Tell me about the information you were provided with concerning the handling of your child's OADs at home’; ‘Tell me if you feel safe when you handle your child's OADs at home’; ‘What do you think about managing your child's drug treatment at home?’; ‘Tell me about the information you were provided with concerning handling your child's bodily excretions at home, e.g. vomit and urine’. The interviews lasted for 8–20 min. All the interviews were audio‐recorded and subsequently transcribed.

### 2.4 Data analysis

Data were analysed using qualitative content analysis. The data analysis started with a naive reading of each interview by all the authors to capture the overall meaning. Notes on the overall impression of each interview were made. With the aim in mind, the interviews were then read through again following an organized format. Meaning units were underlined, condensed and described by a code by the first author. The last author also coded the interviews independently. The codes were sorted into larger sets that eventually formed the categories and subcategories. Discussions between the first and last authors continued during the analytical process. The categories and subcategories were then further discussed by all authors until consensus was reached and the final categories and subcategories were established. Examples of quotes from the parents are shown in Table 2. We used MAXQDA‐plus software (VERBI Software Consult Sozialforschung GmbH Invalidenstraße) to apply codes to the transcripts.^21^

**Table 2:** Parents' statements on handling oral anticancer drugs at home, by category and subcategory

Categories	Subcategories	Example of statements
Parent's views on the provided information	*Lack of, too little or contradictory information*	‘Previously it was more stressful, I thought a lot about the exposure and that he has a twin brother’ (Mother #17) ‘We have found our routine, if it is right or wrong, good or bad, I do not know’(Mother #7) ‘I felt stressful that I was now responsible for my child's drug treatment’ (Mother #43) ‘It is a lot a lot, high demands on the parents, they have to take an active part in this game and have listened and captured all the needed information’ (Mother #57) ‘It is hard to tell, because I was so chocked, so I do not know if I did not understand it or if I was not even listening’ (Mother #5) ‘Ohhh I got really stressed, you know because it is dusty and smoky around this’ (Mother #17) ‘I received very good information when it comes to the oral solution, but when we gave her the tablets it was not so much information about it’ (Mother #9)
	*Parents preference for information delivery*	‘It is never a disadvantage to get additional information, a video to watch… It would further enhance the whole thing especially in the beginning when it feels very unusual and a little nervous, it would feel a little calmer’ (Mother # 26) ‘The most important is also to receive written instructions with all the needed information’ (Mother #19) ‘All the information need to be gathered in one place so it will be easily accessible when needed’ (Mother #32) ‘There are many words that can be difficult for someone who does not speak Swedish, if you had something with pictures or movies or something like that, it would make it easier, so that you can also… not only that you hear Swedish, but that you also see the process: ‘Well, that word means this’. I had to google pretty much in the beginning’ (Mother #5)
Safer over time		‘The tablet form, how we should handle it at home, using gloves and not near food and beverage, we received this information later, when we asked for it’ (Father #28) ‘We are very used to that now and we have learned over time what to do’ (Mother #20).‘I thought it was uncomfortable at first to deal with it because it sounded like it was so dangerous to ingest, and it is also uncomfortable to give it too’ (Mother #21)
Correct drug dose		‘Oral solution that my child keeps in his mouth and then it drops out…. both that you do not know the amount of waste and how much he gets from the dose. It is such small differences that makes such a big difference’ (Father #16) ‘There are always a minimum of 5% that goes to waste’ (Mother #26) ‘The dose was 13.24 mL, it is not visible on the syringe!’ (Mother #20)
Drug intake		‘We took the first tablet here on the ward, a consultant nurse showed us’ now you do like this’ (Mother #42) ‘In the beginning we almost felt that we did abuse our child, that we had to force him. It was very hard’ (Mother #17) ‘I cannot come to the department every day to get help with my child's drug intake. I have to take responsibility and depend on myself’ (Mother #25) ‘I just wanted to shake these nurses:’ help me’, because I felt that they might not be able to help me the way I wanted to’ (Mother #5)

## 3 RESULTS

### 3.1 The experiences of parents handling oral anticancer drugs at home

The parents felt that the period of cancer diagnosis was an overwhelming time for the whole family. At the beginning of the period of illness, the parents reported that they received a lot of information that they had to take in, remember and understand. As time passed by, however, the parents began to adapt to the situation and became more and more involved in the drug treatment of their child. They began to take more responsibility both in hospital and at home. The parents’ ways of taking responsibility were clearly presented through the whole illness trajectory, from ensuring access to the drug from the pharmacy, through double‐checking the provided information by the HCP, to preparing the correct dose and administering the drug to their child. Taking responsibility for the drug treatment of their child was prominent in all the interviews, and the categories that emerged from the analysis all involved this aspect: parents’ views on the provided information—*lack of, too little or contradictory information, and parents’ preferences for information delivery*; safety over time; correct drug dose; and drug administration.

### 3.2 Parents’ views on the provided information

#### 3.2.1 Lack of, too little or contradictory information

Some of the parents knew that anticancer drugs are highly potent medicines that should be handled with care, for example by not touching the drug or by wearing gloves. The parents wanted to know why these drugs should be handled in a specific way and what the risk of exposure was for themselves and for other family members; for example, whether the parent could be affected by the drug when breastfeeding. To actively see the powder scattered widely when the tablets were crushed was described as unpleasant and stressful because of the risk of exposure for themselves and for other family members. Information provided by some physicians that OADs are not as potent as intravenous drugs contributed to a more relaxed way of handling the drug at home. Some parents were aware that anticancer drugs should be handled safely by using protective equipment (e.g., gloves), but they chose to wash their hands with soap and water after administering the drug in the hope that this would be enough. In this respect, some parents were told to use gloves and others were told to wash their hands after handling the drugs at home. This contradictory information from HCPs on handling drugs resulted in parents feeling worried and unsafe.

When parents lacked information, they found their own ways of solving the problem; for example, some used scissors to open capsules or a knife to split tablets. The lack of standardized instructions to inform the parents about their child's drug treatment was also seen in the timing of the delivery of information. The parents described how they needed information at the beginning of their child's oral drug treatment, before taking over responsibility from the HCPs and creating their own routines. When information was lacking, the parents approached other parents, browsed the internet or checked the Summary of Product Characteristics to source the information they needed.

**Figure 1:**
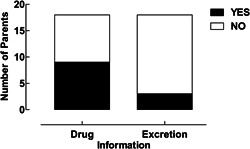
Overview of the number of parents who received information about handling oral anticancer drugs and bodily excretions at home. Black: all parents who answered yes to the questions; white: all parents who answered no to the questions.

During treatment with intravenous anticancer drugs in the hospital, the information that was provided on how to handle bodily excretions was standardized and appeared sufficient. However, only a few parents (17%) received information on bodily excretions before beginning treatment with OADs at home. Only 50% of the parents received information on how to handle OADs at home (see Figure 1).

Some parents requested information on how they could access personal protective equipment so they could handle OADs easily and safely in the home setting. Some parents also wondered if the PPE could be delivered to their homes. Not being supplied with PPE or materials for manipulating the drugs complicated the process of drug handling for most parents; they reused the equipment they had been given until it was broken.

#### 3.2.2 Parents’ preferences for information delivery

Information that was repeated, easily accessible, easy to understand, provided in different formats and in their mother tongue was requested by most parents. Many preferred written instructions and movie clips where the process of drug handling was visually presented to ensure correct interpretation. Information about important side effects and methods of manipulating and administering the drug would ease the process of drug handling at home. To have their drug‐related questions answered and to get feedback from HCPs during the drug handling process was also considered to be beneficial and supportive.

### 3.3 Safety over time

As time passed, most parents adapted to their child's illness, felt safer, and found it easier to take in and process any given information. More and more questions were revealed that parents needed an answer for. With time, the process of drug handling became a daily routine and most children learned to swallow the tablets/capsules. Some parents actively chose not to use drug‐handling precautions, for example, not to wash their hands or use PPE.

### 3.4 Correct drug dose

The parents described how they were clearly aware of the dose and its accuracy. Giving the right dose was seen as extremely important and was linked to the survival of their child. Most parents were also aware of the risk of not achieving an accurate dose during the process of drug manipulation. It was felt that the process of drug handling was complicated if the dose was hard to measure accurately.

### 3.5 Drug administration

Some parents received support from HCPs during the child's first drug intake; this was much appreciated. Other parents had to find a solution to drug administration difficulties by themselves, for example, by using a tablet crusher. Parents felt anxiety and sadness if they had to force their child to take the medicine. They reminded the child about the hard times during treatment or told the child about the consequences of not taking the drug. Giving oral suspensions and medicines through a gastric tube was reported to ease the process of drug administration and to give the parents a sense of relief.

## 4 DISCUSSION

This study showed that parents of children receiving OADs at home expressed a lack of knowledge about the correct handling of OADs and safe drug handling in general, and stated that they had some difficulties in obtaining the correct drug dose for their child and in administering the drug. An interesting observation was that mainly mothers seem to have the responsibility for the treatment of the children.

Parents reported that leaving the hospital after the first treatment of their child's cancer was overwhelming. Their lack of information and feelings of responsibility for the outcome of their child's illness led them to check with the HCPs to ensure that everything was correct. Previous studies have shown that HCPs depend on the parents to care for their child in the hospital and at home from the stage of diagnosis. Parents have to take over the responsibility of caring for their sick child without being prepared.^4^, ^7^, ^22^

Some parents felt that they had received insufficient information, some mentioned contradictory information, and some felt they had received enough information. The receipt of contradictory information gave them a sense of insecurity and mistrust towards the HCP. This finding has been reported in several studies; parents often receive mixed messages from different HCPs, which leads to frustration and confusion. Different HCPs have provided different types of information and parents have learned over time which HCP to ask, depending on the question they have.^1^, ^2^, ^18^, ^22^, ^23^, ^24^

Time has clearly been an important aspect of the study; information given at the right time was the basis of correct drug handling. The lack of standardized information and organized structures affected the timing for the delivery of information. The parents reported that the information given in time helped them to adapt to the information, create a routine and do it right from the beginning. Accurate and timely communication should be prioritized for all parents of children with cancer to increase the parents’ sense of safety and to help them internalize the information provided.^5^, ^7^, ^8^, ^18^

There is limited information about the best methods of delivering education or about the preferences of parents among the available information formats within the overwhelming context of childhood cancer.^5^, ^15^, ^22^, ^24^, ^25^ In this study, parents reported that being provided with information in several different formats, and in their mother tongue, was preferable. Several studies have described different methods that can be used to help parents learn, recall and understand the relevant information. These have emphasized the importance of enhancing the parents’ self‐sufficiency by combining different information formats; for example, oral information should be accompanied by written information and a follow‐up contact by a nurse. Movie clips can also be used to educate parents on complicated topics.^1^, ^5^, ^23^, ^25^, ^26^, ^27^, ^28^

Parents from non‐Swedish‐speaking backgrounds expressed their wish to be provided with information in their mother tongue so as to be able to fully understand and process the information. It is well known that parents from different ethical backgrounds are often underinformed by HCPs and that both language ability and culture can be barriers to understanding the provided information.^1^, ^4^, ^29^, ^30^

It is believed that some HCPs withhold information from parents to protect them from anxiety and distress. In our study, only 17% of the parents were informed about handling bodily excretions in the home setting. Paternalism in medicine has been well described; the implication is that HCPs make decisions based on what they determine to be in the patient's/parents’ best interests. There is a need to increase HCP awareness about the risks of incorrect handling of OADs and the need for provision of consistent and standardized information for parents.

The parents in this study were clearly aware of two aspects of drug handling: obtaining the correct dose and ensuring drug intake. Drug administration was experienced as problematic; it affected not only themselves as parents but also their children. There is a need to help both parents and their children with drug intake through practical drug‐administration training and support. It is also important to include the children as active participants in their own drug treatment, to help them with drug intake and to ease the feelings of guilt in their parents.

### 4.1 Methodological reflections

This study has some strengths that should be considered. It included the parents of children with varied diagnoses, ages, drug treatments, drug forms and lengths of treatment. This enriched the variety of data, strengthening credibility. Although only a few drugs were included, all manipulation procedures were included. The study investigated areas about which there has historically been little information: the parents’ experience of handling OADs in a home setting and the parents’ preferences among the available information formats.

All the authors worked together in the analytical process to minimize the risk of pre‐assumptions and to strengthen the credibility and confirmability of the study. While the first, second and last authors had close dialogues during the entire process, they also coded some of the interviews independently of each other. Trustworthiness was further enhanced by selecting quotations from some parents.

The study also has some limitations: the sample size was small, it included only a few parents of children with brain tumours, and only one paediatric oncology centre was involved. The trustworthiness of the interviews might have been influenced by the fact that only one parent per child was interviewed on only one occasion. In addition, parents were interviewed on different time occasions during the treatment period, often lasting several years.

## 5 CONCLUSIONS AND IMPLICATIONS FOR PRACTICE

Accurate, timely, nonconflicting, repeated information should be given to parents in different formats and languages about how to handle OADs at home. Parents need to be informed about the risks associated with handling OADs and to be supplied with the correct equipment to motivate correct drug handling at home. Parents need support in drug administration from HCPs and the child should also be included in learning about their own drug treatment to facilitate drug intake and increase feelings of safety.

## CONFLICT OF INTEREST

The authors declare no conflict of interest.

### 1 ETHICS STATEMENT

The study was approved by the Regional Ethical Review Board in Stockholm (DNR: 2019‐03192). Oral and written information about the purpose of the study, confidentiality and the right to discontinue their participation at any time was provided to all participants. Participants were also asked to sign a consent form before the interviews began.

## Supporting information

Supplementary information.

## Data Availability

Data sharing not applicable—no new data generated, or the article describes entirely theoretical research.
