# Supplementary material for: Parents' experiences of handling oral anticancer drugs at home: ‘It all falls on me …’
Source: J Eval Clin Pract. 2022 Aug 4;29(1):94–100. doi: 10.1111/jep.13737 (PMC10086976; doi:10.1111/jep.13737)
Supplement: Supplementary file 1 — Supplementary information. [file JEP-29-94-s001.pdf]

Appendix 1

Interview guide

1. Tell me about the information you were provided with concerning the handling of your child's oral anticancer drugs at home?
2. Where did you receive the information?
3. How did you experience the information you received?
4. What can be improved?
5. Where do you seek information on medication management if needed?
6. Is there any information that are missing?
7. In what forms would you like to receive information about drug management?
8. What do you think about managing your child's drug treatment at home? What do you think works well/less? Please describe.
9.Tell me if you feel safe when you handle your child's oral anticancer drugs at home?
10. How do you manipulate the medicine (eg crush / split / dissolve a tablet or open a capsule)? Please describe.
12. Do you experience any difficulties in manipulating the medicine? Please describe
13. What information have you been given about the equipment you will need to use to manipulate the medicine?
14. Tell me about the information you were provided with concerning handling your child's bodily excretions at home, e.g. (Examples vomit and urine)?
